# Control of Algae-Induced Disinfection By-Products Formation by Pre-Oxidation for Typical Algal Species in Drinking Water Treatment Plants of Chongqing, China

**DOI:** 10.3390/toxics14090826

**Published:** 2026-09-17

**Authors:** Xiangyu Chen, Shuhan Xia, Hao Zhong, Dan Gong, Haibing Cong

**Affiliations:** 1 College of Environmental Science and Engineering, Yangzhou University, Yangzhou 225127, China; 2 Key Laboratory of the Three Gorges Reservoir Regions Eco-Environment, Ministry of Education, Chongqing University, Chongqing 400045, China

**Keywords:** algae-laden water, Potassium permanganate pre-oxidation, ozone pre-oxidation, integrated control process, algae-derived disinfection by-products

## Abstract

Severe eutrophication and frequent algal blooms in source waters (the main stream of the Yangtze River, the Jialing River tributary, and the Three Gorges Reservoir area) pose significant challenges to water treatment plants (WTPs) in Chongqing Municipality, China. This leads to reduced efficiency of conventional treatment and elevated disinfection by-products formation potential (DBPs-FP) in high algae-laden water. This study systematically evaluated the algae removal efficacy of pre-oxidation using potassium permanganate (KMnO_4_) and ozone (O_3_), alongside their control effects on algae-derived DBPs-FP, targeting two typical bloom-forming species: the typical cyanobacteria, i.e., *M. aeruginosa* and the typical diatom, i.e., *Synedra* sp. Results demonstrated that both low-dose O_3_ and micro-acidified low-dose KMnO_4_ effectively inactivated both algal species. Based on pre-treatment efficacy, targeted integrated control processes compatible with existing Chongqing WTP infrastructure were proposed. The combination of micro-acidified low-dose KMnO_4_–powdered activated carbon (PAC)–conventional water treatment process was effective in controlling *M. aeruginosa*-derived DBPs. Conversely, the integrated sequence of low-dose O_3_ pre-oxidation–conventional treatment–O_3_–Biological Activated Carbon (BAC) was highly effective for controlling *Synedra* sp.-derived DBPs. This research provides crucial technical support and a parametric basis for optimizing treatment processes in Chongqing WTPs handling high algae-laden source waters, specifically for mitigating algae-derived disinfection by-products.

## 1. Introduction

Harmful algal blooms are a major and serious environmental issue because of their negative effects on aquatic ecosystems and human health [1,2]. Cyanobacteria and diatoms are well-known algal species and are widely distributed in almost all aquatic habitats, and their blooms can change water quality and cause problems with its treatment [3,4,5]. Chongqing, a pivotal water hub in the upper reaches of the Yangtze River, relies predominantly on the mainstream of the Yangtze River. The Jialing River tributary and the Three Gorges Reservoir system serve as its drinking water source. The operational pattern of the Three Gorges Reservoir, characterized by “storing clear water and discharging turbid water,” has reduced flow velocities within the Chongqing section of the Jialing River and its reservoir tributaries [6,7]. This hydrological change promotes the continuous accumulation of nitrogen- and phosphate-containing nutrients, thus exacerbating the eutrophication levels in reservoir bays [8,9,10]. Seasonal algal blooms are a recurrent issue. Diatom-dominated blooms (e.g., *Synedra* sp.) frequently occur in spring and summer [11,12], whereas cyanobacteria-dominated blooms (e.g., *Microcystis aeruginosa*) are prevalent in autumn and winter [13,14]. These phenomena pose multi-faceted threats to drinking water security.

High algal concentrations in source water could impair the efficiency of conventional water treatment processes [15,16]. Wang et al. reported that during severe blooms in the tributaries of the Three Gorges Reservoir, cell densities of typical algae frequently reach 10^7^–10^8^ cells/L [13]. Zhou et al. similarly documented such bloom cell densities in the Chongqing section of the Yangtze River [17]. Furthermore, upon cell death or lysis, algae will release substantial amounts of algal organic matter (AOM) [18,19]. When this AOM flows into water treatment systems, it can act as a precursor and react with chlorine during the disinfection process to form various disinfection by-products (DBPs), including trihalomethanes (THMs), haloacetic acids (HAAs), and haloacetonitriles (HANs), which could significantly impact the water quality [20,21].

Most WTPs in Chongqing currently employ conventional treatment processes, typically comprising coagulation, sedimentation, filtration, and chlorination. These processes demonstrate limited effectiveness in controlling algae-derived DBPs [22,23]. To address challenges posed by high algae-containing source waters, permanganate (KMnO_4_) pre-oxidation or ozone (O_3_) pre-oxidation are two prevalent technologies adopted in water treatment [24]. KMnO_4_ pre-oxidation under micro-acidified conditions shows significant efficiency in inactivating both cyanobacteria and diatoms. However, overdosing can lead to manganese ion deposition and potentially cause “black water” issues in distribution networks [25]. O_3_ pre-oxidation can efficiently destroy algal cell structures but involves relatively higher operational costs, and may promote the release of intracellular organic matters [26,27].

In the advanced water treatment process, the synergistic application of powdered activated carbon (PAC) and the combination of ozone–biologically activated carbon (O_3_–BAC) advanced treatment process can significantly enhance the removal of organic matter [28,29]. PAC exhibits optimal adsorption for organic compounds within the molecular weight range of 500–1000 Da [30]. The O_3_–BAC process firstly leverages ozonation to break down complex organic molecules and increase their biodegradability. The subsequent adsorption and biodegradation on the activated carbon filter can achieve the removal efficiencies of THM and HAA precursors up to 70% and 50%, respectively [31]. Despite these advancements, previous studies focused on individual unit processes or single oxidants, but a systematic evaluation of the sequential integration of pre-oxidation, conventional treatment, and advanced processes (PAC or O_3_–BAC) is lacking, especially for site-specific dominant species in Chongqing. There remains a scarcity of systematic investigation into the degradation pathways of algae-derived AOM and the resultant DBPs-FP under integrated treatment schemes that combine pre-oxidation, conventional treatment, and advanced treatment.

Given the seasonal succession of dominant algal species in source water of Chongqing and the inherent limitations of conventional processes, the precise control of algae-derived DBPs through the synergistic application of KMnO_4_/O_3_ pre-oxidation with complementary technologies like PAC or O_3_–BAC becomes paramount for ensuring water supply safety. This study aims to utilize typical dominant bloom-forming algal species from source water of Chongqing, and systematically investigate the release patterns of AOM and the formation mechanisms of DBPs under different pre-oxidation processes. Most works report only bulk DBPs-FP reduction, without addressing how pre-oxidation qualitatively changes precursor properties. In this study, we used the removal contribution of each sequential unit to infer these transformations. The ultimate objective of this study is to propose and validate specifically designed and efficient integrated water treatment processes for controlling algal blooms in source waters.

## 2. Materials and Methods

### 2.1. Materials and Algal Cultivation

Two typical cyanobacteria and diatom algal species, i.e., *Microcystis aeruginosa* (*M. aeruginosa*, strain FACHB-315) and *Synedra* sp. (strain FACHB-1296), were selected for this study. They were obtained from the Freshwater Algae Culture Collection at the Institute of Hydrobiology (FACHB), Chinese Academy of Sciences. Stock cultures of each algal species were maintained under standard laboratory conditions. Briefly, *M. aeruginosa* and *Synedra* sp. were cultivated in BG11 and AGP medium, respectively. The complete compositional details of each medium following the FACHB standard are provided in Tables S1 and S2, Supplementary Materials. All cultures were maintained in a temperature-controlled incubator at 25 ± 1 °C under a 12 h:12 h light:dark photoperiod with an illumination intensity of 2000 lux. For experimentation, algal suspensions were diluted using sterile medium and the initial cell density used for each experiment was confirmed to be 1.0 × 10^8^ cells/L, simulating conditions of a representative severe algal bloom. Briefly, algal cultures were harvested at mid-to-late exponential phase (days 10–14), confirmed by daily cell counts. Cell density was determined using a Neubauer improved haemocytometer (0.1 mm depth) under an optical microscope at 400 × magnification, with at least three counting replicates per sample. The average value was calculated using the formula: Cell density (cells/L) = (average cell count per grid) × dilution factor × 10^4^/(number of grids counted). The pH value of diluted algal suspensions was meticulously adjusted to the desired values using either 1 mol/L NaOH or 0.1 mol/L HCl solution.

For each experimental run, a 200 mL aliquot of the pH-adjusted algal suspension was transferred into a 250 mL stoppered glass iodine flask containing a small, clean magnetic stir bar. The flask was then securely positioned in a temperature-controlled water bath equipped with a magnetic stirring plate. The stirring speed was maintained constant at 200 rpm for 1 h, and the temperature was controlled at 25 ± 1 °C throughout the experiments unless otherwise specified.

### 2.2. Experimental Methods

#### 2.2.1. Conventional Water Treatment Simulation

All experiments were performed in three independent biological replicates (different culture batches), each with two analytical duplicates. An initial algal density of 1 × 10^8^ cells/L was maintained uniformly, as verified by microscopic counting prior to each treatment. All data are presented as mean ± standard deviation (SD, *n* = 3). Jar-test experiments were conducted to simulate conventional water treatment processes and evaluated their effectiveness in controlling DBPs-FP for the two target algal species. The experimental procedure was as follows: Algal suspension with an initial concentration of 10^8^ cells/L served as the synthetic raw water. A 150 mL sample of this suspension was first subjected to pretreatment involving centrifugation at 8000 rpm for 10 min, followed by vacuum filtration through a 0.45 μm pore-size glass fiber membrane. This pretreated sample was used to determine the initial DBPs-FP prior to any treatment simulation.

Coagulation experiments were performed using a standard six-paddle jar-test apparatus. The programmed stirring sequence consisted of an initial rapid mix phase at 200 rpm (corresponding to a mean velocity gradient, G-value, of approximately 452 s^−1^) for 1 min, immediately followed by a slow mix phase at 50 rpm (G-value ≈ 84 s^−1^) for 15 min. After mixing, the jars were allowed to settle quiescently for 15 min. The coagulant used was ferric chloride (FeCl_3_), applied at a dosage of 10 mg/L (expressed as Fe^3+^). The turbidity of the synthetic raw water was adjusted to approximately 20 NTU using kaolin clay. Following the settling period, 150 mL of the supernatant was carefully collected. This supernatant was then filtered through a 0.45 μm glass fiber membrane, and the filtrate was analyzed to determine the DBPs-FP after the conventional treatment simulation.

#### 2.2.2. KMnO_4_ Pre-Oxidation Experiments

For the algal photosynthetic activity assessment, a predetermined volume of a KMnO_4_ stock solution (0.5 g/L) was added to the iodine flask containing the algal suspension. The flask was immediately stoppered, and the reaction proceeded under constant stirring. After the designated reaction time, the oxidation process was quenched by adding a stoichiometric excess of sodium thiosulfate (Na_2_S_2_O_3_·5H_2_O) stock solution (2 mol/mol KMnO_4_). A blank control, identical in all aspects except for the addition of KMnO_4_, was run concurrently. Immediately upon quenching the reaction, a 10 mL aliquot of the well-mixed suspension was withdrawn for the assessment of photosynthetic activity.

**Figure 1:**
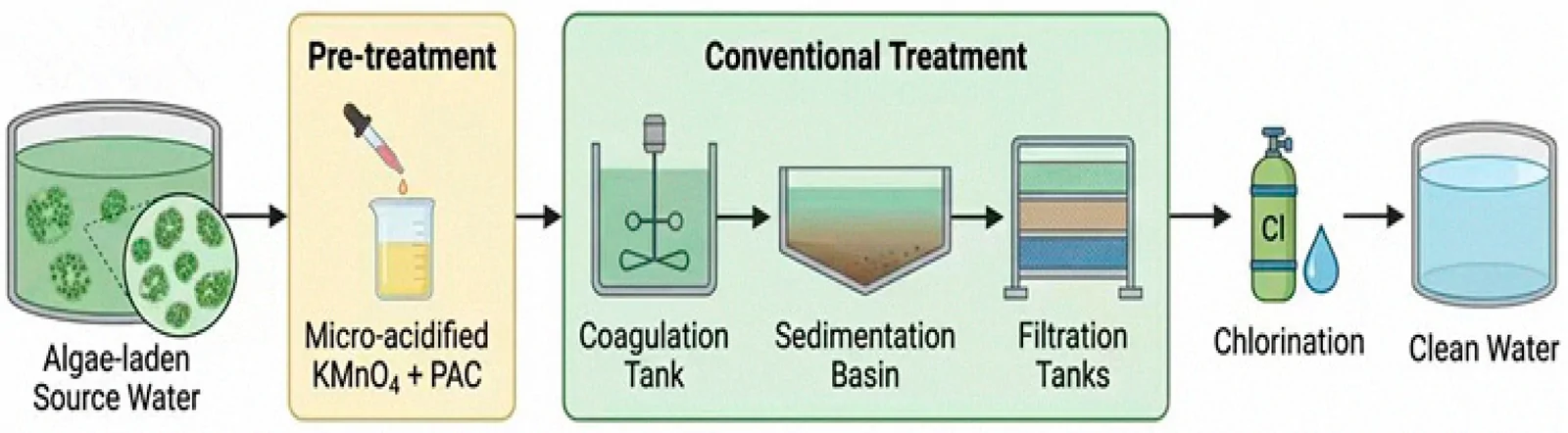
KMnO_4_–PAC–Conventional Treatment integrated process.

The DBPs-FP measurement was conducted as follows: KMnO_4_ pre-oxidation together with PAC adsorption was conducted in 1 L cylindrical beakers placed on the six-paddle stirrer, replacing the smaller iodine flasks. PAC was added simultaneously with the KMnO_4_ stock solution at the beginning of the rapid mix period. All other experimental conditions and steps remained consistent with the photosynthetic activity assays. After the combined pre-treatment, 150 mL algal suspension was centrifuged at 8000 rpm for 10 min. The resulting supernatant was filtered through a 0.45 μm membrane, and the filtrate was used for DBPs-FP testing. The remaining treated suspension was subsequently subjected to the conventional treatment simulation as described in Section 2.2.1. The schematic diagram of the combined KMnO_4_–PAC–conventional treatment process is presented in Figure 1.

#### 2.2.3. O_3_ Pre-Oxidation Experiments

The algal photosynthetic activity assessment during O_3_ pre-oxidation was conducted as follows: A calculated volume of a saturated ozone stock solution, prepared by bubbling high-purity oxygen-fed ozone through ultrapure water cooled in an ice bath and standardized by the indigo method, was pipetted directly into the algal suspension. Following the specified reaction duration, residual ozone in the reaction vessel was stripped by bubbling high-purity nitrogen gas through the suspension for a fixed time to terminate the reaction. Immediately after ozone quenching, a 10 mL sample was taken for photosynthetic activity measurement. A blank control was also included, identical in all aspects except for the addition of O_3_.

**Figure 2:**
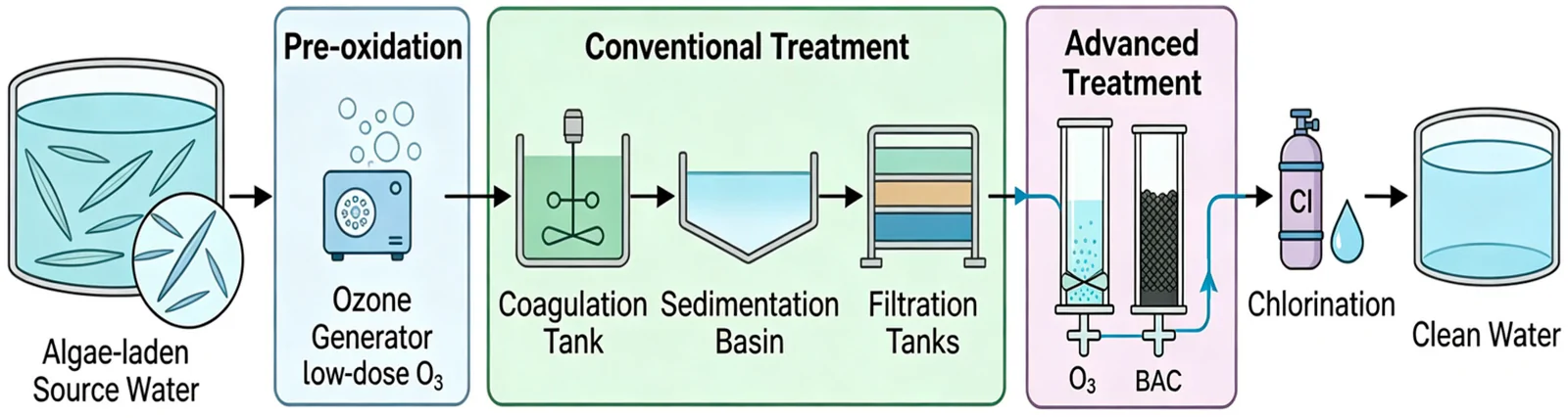
O_3_ Pre-oxidation–Conventional Treatment–O_3_–BAC integrated process.

The DBPs-FP measurement for evaluating the O_3_ pre-oxidation combined with the BAC process mirrored that used for the DBPs-FP tests during KMnO_4_-PAC in terms of reactor configuration and sampling procedures. O_3_ pre-oxidation was followed by the conventional treatment simulation. Subsequently, a post-ozonation step (if part of the specific integrated scheme) was applied, followed by passage through a laboratory-scale BAC column. The BAC contact time was carefully controlled. The schematic diagram of the combined O_3_ Pre-oxidation–Conventional Treatment–O_3_–BAC process is shown in Figure 2.

### 2.3. Analytical Methods

#### 2.3.1. Assessment of Algal Cell Photosystem II (PSII) Activity

The physiological status, viability, and photosynthetic activity of the algal cells were assessed by measuring key parameters related to Photosystem II (PSII) activity using a Phytoplankton Analyzer (PHYTO-PAM, Walz, Effeltrich, Germany). This instrument employs Pulse-Amplitude-Modulation (PAM) fluorometry, a technique based on the measurement of chlorophyll-a fluorescence, which is a highly sensitive and non-destructive tool for characterizing the activity and performance of the photosynthetic apparatus [32]. Four typical parameters were determined through a PHYTO-PAM analyzer, i.e., Y (dimensionless), α (μmol electrons m^−2^ s^−1^/μmol photons m^−2^ s^−1^), and rETR_max_ (μmol m^−2^ s^−1^), along with the concentration of chlorophyll-a (μg L^−1^) directly. The detailed determination and computation of the above photosynthetic parameters were presented in Text S2, Supplementary Materials.

Photosynthetic Yield (Y) represents the effective quantum yield of PSII photochemistry. It is a direct indicator of the algal “growth potential” and instantaneous photosynthetic capacity. A decrease in Y value suggests impaired light harvesting and/or inhibition of the photosynthetic electron transport chain [33]. Chlorophyll-a Concentration (Chl-a) indicates the primary photosynthetic pigment and energy transfer medium in PS II [34]. The concentration of chlorophyll-a was directly read by the PHYTO-PAM using its built-in fluorescence-based quantification algorithm, which has been pre-calibrated against standard chlorophyll-a extracts. This indicator serves as a proxy for algal biomass in the suspension.

By exposing the algae to a series of rapidly increasing actinic light intensities and measuring the corresponding fluorescence response, a rapid light curve (RLC) was generated. Fitting the RLC provided two key parameters, i.e., initial slope (α) and maximum relative electron transport rate (rETR_max_). The α of the RLC (units: μmol electrons m^−2^ s^−1^/μmol photons m^−2^ s^−1^) reflects the light utilization efficiency of the photosystems [35]. A decline in α indicates damage to the photosynthetic system, specifically affecting energy transfer and light capture capabilities. The rETR_max_ (units: μmol electrons m^−2^ s^−1^) derived from the RLC represents the theoretical maximum capacity of PSII electron transport. A reduction in rETR_max_ signifies inhibition of the electron transport chain within PSII [36].

In this study, the above four typical parameters were collectively used to comprehensively reflect algal cell viability and the extent of photosynthetic inactivation resulting from pre-oxidation treatments. Of note, the term “photosynthetic inactivation” is operationally defined as an irreversible severe impairment of the PSII electron transport chain, as indicated by near-zero values of the above four typical parameters. We acknowledge that this does not directly confirm loss of membrane integrity or cell death; however, it is widely used as a sensitive proxy for physiological damage in phytoplankton studies.

#### 2.3.2. Determination of DBPs-FP Formation Potential

The formation potential of disinfection by-products was assessed using a standardized chlorination incubation procedure. After the respective treatment processes (conventional alone or integrated schemes), the filtered water samples (through 0.45 μm) were buffered to pH 7.0 ± 0.2 using a 0.05 M phosphate buffer. NaOCl stock solution (~5% active chlorine) was freshly prepared and standardized by the DPD method. The dose was pre-determined by chlorine demand tests for each sample to achieve a free chlorine residual of 1.0 ± 0.2 mg/L after 7 d at 25 °C in the dark. Residual chlorine was measured before and after incubation using a Hach DR6000 spectrophotometer (HACH LANGE GmbH.: Berlin, Germany) and was then quenched using ascorbic acid. The concentrations of specific DBPs, namely, trihalomethanes (THMs), haloacetic acids (HAAs), and haloacetonitriles (HANs) in the quenched samples were determined using a Gas Chromatograph (GC-2010plus, Shimadzu, Kyoto, Japan) equipped with an electron capture detector (ECD) following standard USEPA methods or their equivalents (e.g., EPA 551.1 for THMs/HANs, EPA 552.3 for HAAs). More detailed procedures for determining DBPs-FP were described in Text S1, Supplementary Materials. The DBPs-FP for each species was reported as the concentration measured after incubation. The representative GC-ECD chromatograms of the standard mixture were provided in Figures S1–S3, Supplementary Materials. Also, the calibration curves, which illustrate the linear relationship between the concentration of DBPs and their corresponding peak area as determined by GC-ECD, are presented in Figures S4–S6, Supplementary Materials.

It should be emphasized that the DBPs-FP values reported here represent the maximum potential formation under exaggerated chlorination conditions and are not direct measurements of DBP concentrations in finished drinking water. The comparison with regulatory limits is intended as a conservative, worst-case assessment to evaluate precursor removal efficiency and to provide a safety margin for process design. Actual finished water DBP concentrations in full-scale plants would be expected to be lower than the DBPs-FP values due to shorter contact times, lower residual chlorine levels, and other operational factors.

## 3. Results and Discussion

### 3.1. Photosynthetic Inactivation of Typical Algal Species by KMnO_4_ Pre-Oxidation Alone

#### 3.1.1. Photosynthetic Inactivation of *M. aeruginosa*

**Figure 3:**
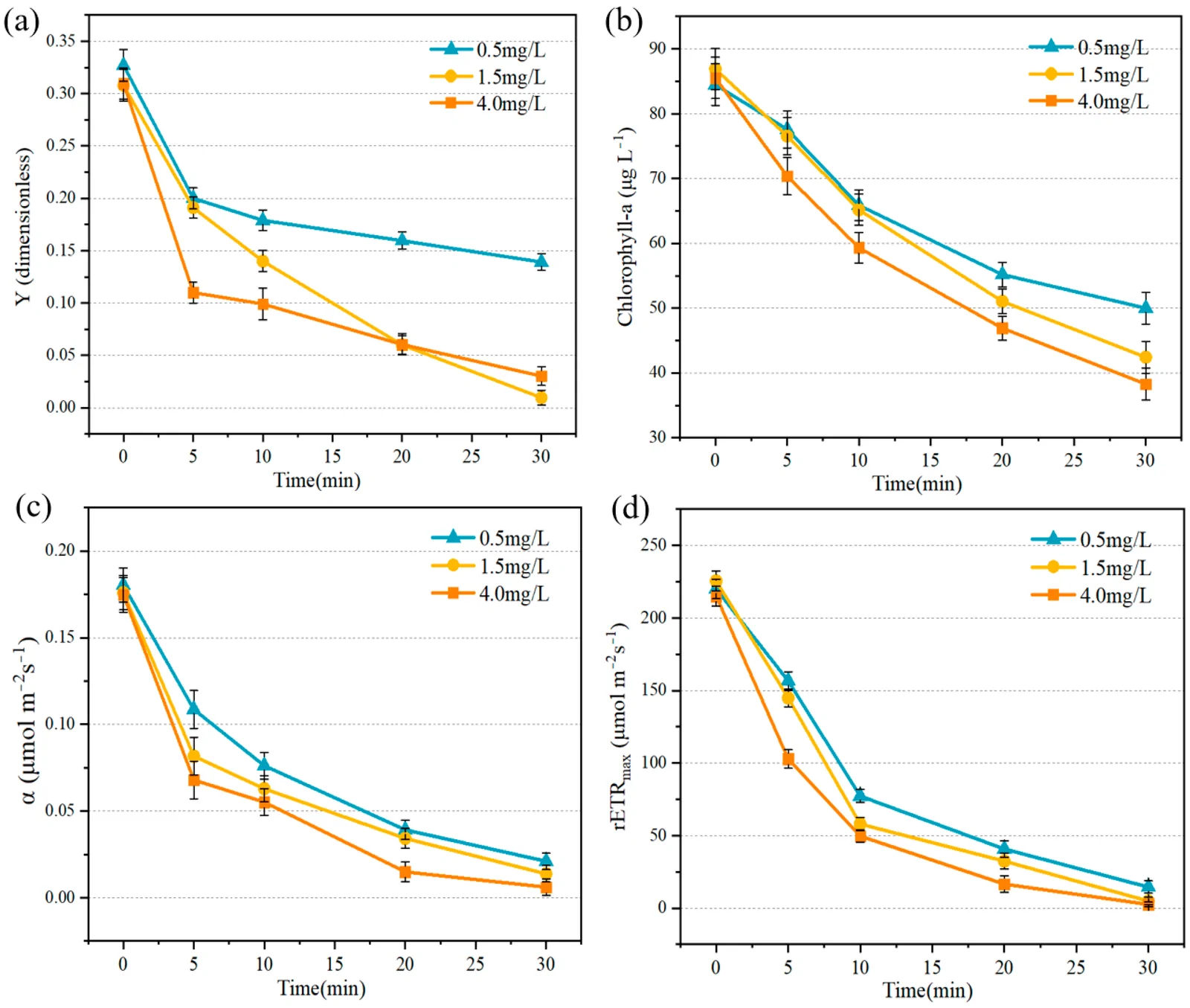
Photosynthetic inactivation effects of different doses of KMnO_4_ on *M. aeruginosa* at pH = 5.0: (**a**) Y, effective quantum yield; (**b**) concentration of chlorophyll-a; (**c**) α, initial slope; (**d**) rETR_max_, maximal electron transport rate.

The oxidative efficiency of KMnO_4_ is known to be pH-dependent, exhibiting a higher redox potential under acidic conditions (E_acid_ = 1.69 V) compared to neutral (E_neutral_ = 0.588 V) or alkaline (E_alkaline_ = 0.564 V) environments [26]. Therefore, this study specifically investigated the impact of KMnO_4_ pre-oxidation on the photosynthetic system of *M. aeruginosa* within an acidic regime (pH = 5.0). The experimental results depicted in Figure 3 revealed a pronounced declining trend in all the key photosynthetic parameters (Y, chlorophyll-a, α, and rETR_max_) following a 30 min oxidation treatment.

When the KMnO_4_ dosage was increased to 1.5 mg/L, the values of Y, α, and rETR_max_ approached the instrumental detection limit (i.e., near zero), while the chlorophyll-a concentration also exhibited a significant reduction exceeding 50%. Notably, the algal photosynthetic inactivation efficiency observed for the 1.5 mg/L and 4 mg/L dosage groups was quite similar, and both were markedly superior to the performance of the 0.5 mg/L dosage group. This observed dose–response relationship aligns well with findings previously reported by Knappe et al. regarding various algae inactivation by KMnO_4_ [37].

The mechanism of algal cell photosynthetic inactivation by KMnO_4_ pre-oxidation primarily involves its ability to penetrate the cell wall, thereby reducing photosynthetic capacity and ultimately leading to cell lysis [38]. Concurrently, Chen et al. observed that KMnO_4_ can promote the aggregation of algal cells. The flocs formed by these aggregated cells can provide a protective effect to some extent, mitigating the destructive action of KMnO_4_ on individual cells within the aggregate [39]. This phenomenon likely explains the initial rapid decline of photosynthetic parameters observed within the first 10 min of reaction, followed by a subsequent slowing of the inactivation rate. Considering the potential secondary pollution issues associated with excessive KMnO_4_ dosing, such as elevated effluent turbidity, abnormal color, and excessive total manganese concentrations, and balancing technical feasibility with economic and risk management considerations for full-scale water treatment plants, a dosage of 1.5 mg/L was selected as the optimal KMnO_4_ pre-oxidation dose for subsequent integrated process experiments.

#### 3.1.2. Photosynthetic Inactivation of *Synedra* sp.

**Figure 4:**
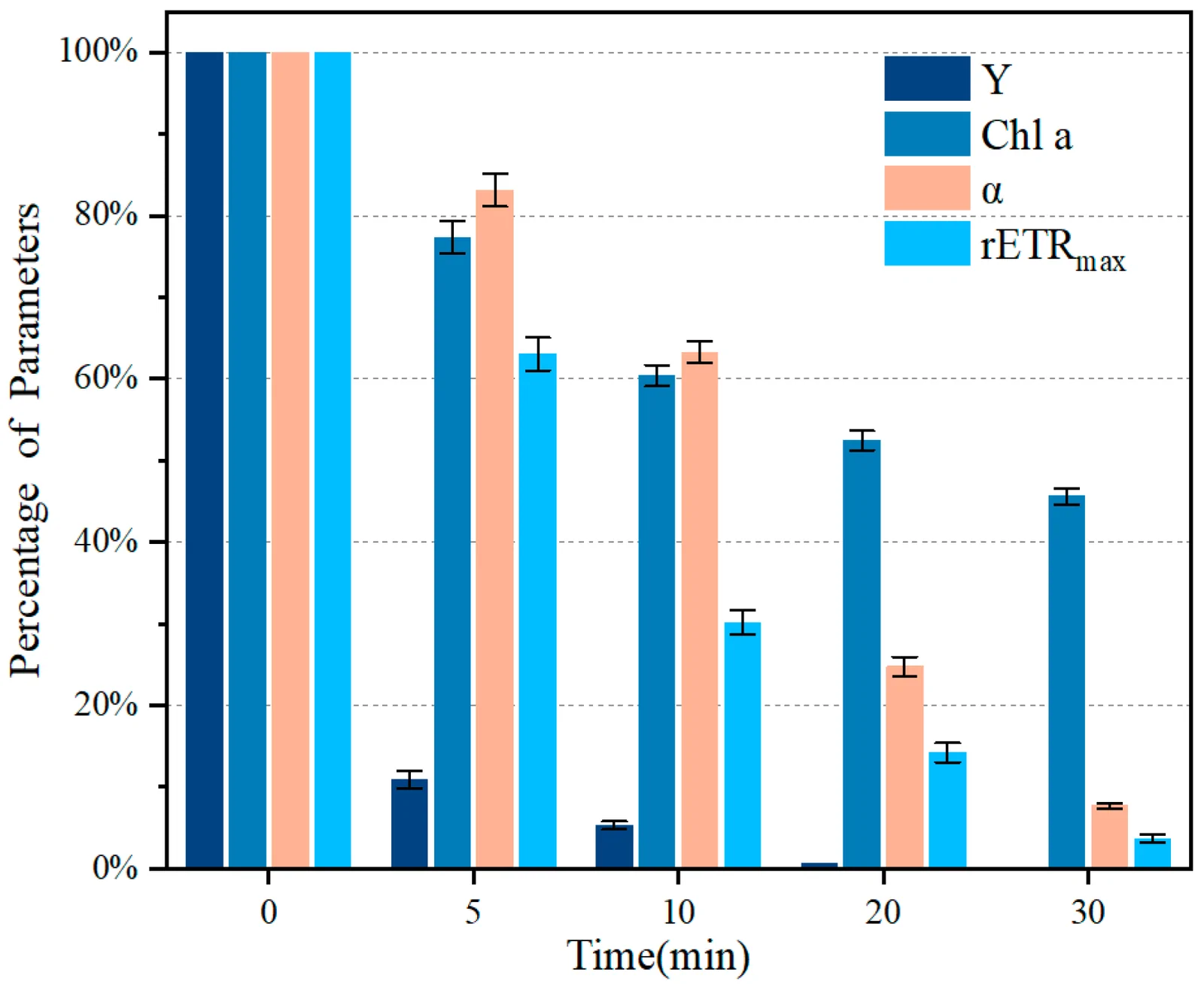
Photosynthetic inactivation effect of KMnO_4_ on *Synedra* sp. at pH = 5.0.

A micro-acidified system (pH = 5.0) was selected in this study to investigate KMnO_4_ pre-treatment effects on *Synedra* sp. This selection was based upon empirical research by Zhang et al., which demonstrated the pH-dependent efficiency of KMnO_4_ for inactivating *Synedra* sp., with an efficiency order of pH = 5.0 > pH = 7.0 > pH = 9.0 [40]. As illustrated in Figure 4, under these micro-acidic conditions, a KMnO_4_ dose of 1.5 mg/L exhibited highly effective photosynthetic inactivation characteristics against *Synedra* sp. at the high bloom density (10^8^ cells/L). After just 15 min of treatment, the Y value had decreased below the detection limit. Upon extending the reaction time to 30 min, both the α of the rapid light-response curve and the rETR_max_ were significantly reduced and approached 0. Concurrently, the concentration of the biomarker chlorophyll-a also decreased by over 50%.

Under identical pre-oxidation conditions (pH = 5.0, 1.5 mg/L KMnO_4_, 30 min), *Synedra* sp. displayed greater sensitivity to KMnO_4_ compared to *M. aeruginosa*. A plausible explanation for this differential sensitivity lies in their distinct cellular structures. *Synedra* sp. typically exists as single cells or short chains characterized by a fragile cell wall composed primarily of bilayered silica [41]. In contrast, *M. aeruginosa* often forms colonial structures encased in a robust cell wall consisting of peptidoglycan and an outer polysaccharide glycocalyx or sheath. These high-molecular-weight components in *M. aeruginosa* can significantly enhance resistance to external oxidative stress [42]. Consequently, *Synedra* sp., with its more delicate siliceous frustule, is more readily inactivated during KMnO_4_ pre-oxidation compared to the sheathed and colonial *M. aeruginosa*.

### 3.2. Photosynthetic Inactivation of Typical Algal Species by O_3_ Pre-Oxidation Alone

#### 3.2.1. Photosynthetic Inactivation of *M. aeruginosa*

**Figure 5:**
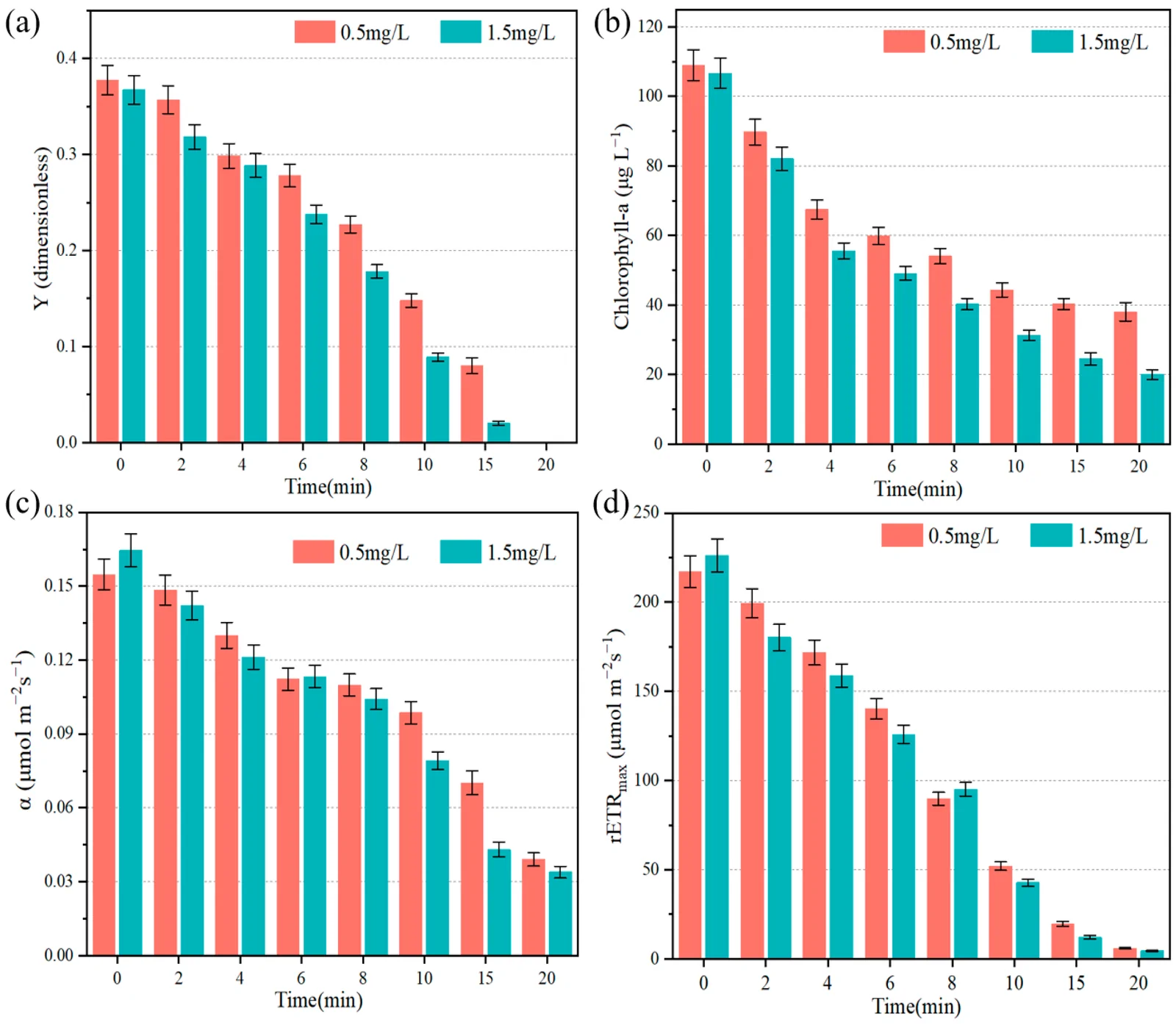
Photosynthetic inactivation effects of different doses of O_3_ on *M. aeruginosa*: (**a**) Y, effective quantum yield; (**b**) concentration of chlorophyll-a; (**c**) α, initial slope; (**d**) rETRmax, maximal electron transport rate.

O_3_ Pre-oxidation was performed by adding calculated volumes of a saturated ozone water solution to the suspensions of *M. aeruginosa* (10^8^ cells/L) to evaluate photosynthetic inactivation efficiency and establish dose–response relationships. Experimental results shown in Figure 5 indicated that both the 0.5 mg/L and 1.5 mg/L O_3_ doses rapidly and effectively inactivated *M. aeruginosa*, with the 1.5 mg/L treatment group demonstrating superior efficiency. After 15 min of oxidation with 1.5 mg/L O_3_, the Y and rETR_max_ plummeted to values near 0. The Chlorophyll-a concentration and the α also decreased to levels close to their minimum detectable values, confirming that this oxidation process achieved effective photosynthetic inactivation of *M. aeruginosa*. Research by Wang et al. also indicated that the inactivation rate of *M. aeruginosa* noticeably increased with higher O_3_ doses (0.5–3 mg/L) and longer contact time (0–40 min) [43]. Findings from Wu et al. showed that at a fixed ozonation time of 30 min, the algal density of *M. aeruginosa* decreased with increasing O_3_ dose [44]. However, when the O_3_ dose exceeded 4 mg/L, the algal density scarcely decreased further, plateauing at a photosynthetic inactivation rate of around 40%. In the present study, using a lower dose of 1.5 mg/L O_3_ and a shorter contact time of 15 min proved sufficient to severely impair photosynthesis, as evidenced by Y and rETR_max_ dropping to 0. At the same time, chlorophyll-a and α value had also reached a very low level. The differences between our results and those of Wu et al. can be explained by differences in water matrix composition. Wu et al. conducted experiments in river water containing background organic matter and inorganic scavengers that would consume a significant portion of the applied ozone. In contrast, our study used a pure algal suspension in synthetic medium, meaning that nearly all applied ozone was available to act on the algal cells. Thus, the effective ozone dose per cell was substantially higher in our system, leading to greater inactivation efficiency at a lower nominal dose. In summary, low-dose O_3_ pre-oxidation can effectively inactivate high-density *M. aeruginosa*.

Under the influence of O_3_, the cell walls of *M. aeruginosa* could undergo swelling and become loosened. Subsequently, the structural integrity of both the cell wall and cell membrane is compromised, with these changes becoming more pronounced at higher O_3_ doses [45]. The underlying mechanism likely involves direct attack by O_3_ molecules and indirect oxidation by potent secondary oxidants, primarily hydroxyl radicals (**·**OH), which are generated from O_3_ decomposition in water. These oxidants target components of the cell wall and membrane, disrupting their integrity and leading to the release of intracellular organic matter (IOM) [46].

#### 3.2.2. Photosynthetic Inactivation of *Synedra* sp.

**Figure 6:**
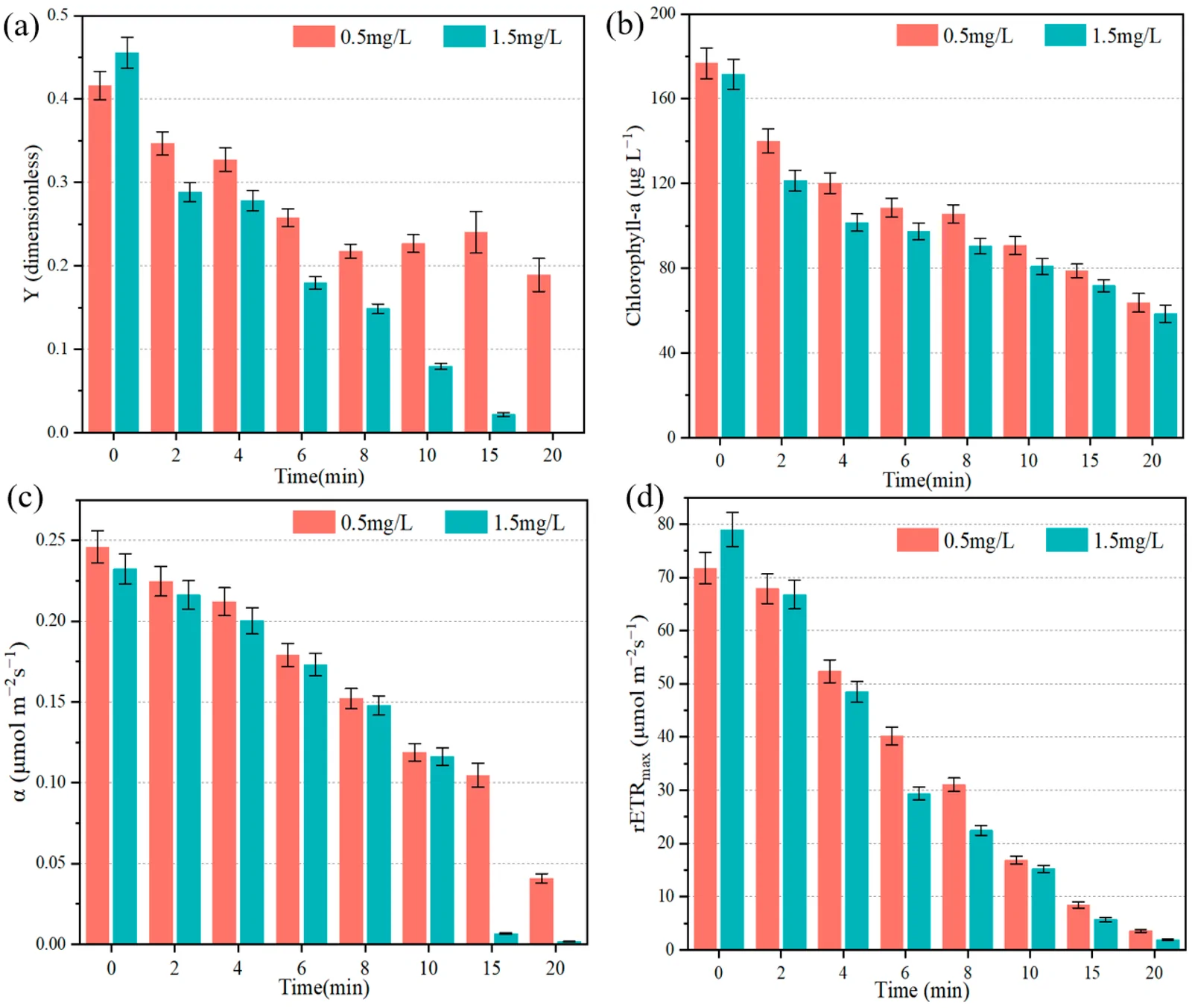
Photosynthetic inactivation effects of different doses of O_3_ on *Synedra* sp.: (**a**) Y, effective quantum yield; (**b**) concentration of chlorophyll-a; (**c**) α, initial slope; (**d**) rETRmax, maximal electron transport rate.

Similarly, low doses of saturated ozone water (0.5 mg/L and 1.5 mg/L) were used to investigate the inactivation efficiency of O_3_ pre-treatment on *Synedra* sp. at the high bloom concentration. Results presented in Figure 6 demonstrate that O_3_ pre-treatment at both low doses can effectively inactivate *Synedra* sp., with the 1.5 mg/L dose yielding superior results. After 15 min of pre-oxidation with 1.5 mg/L O_3_, the Y, α, and rETR_max_ of *Synedra* sp. were essentially reduced by over 90%, and the chlorophyll-a concentration decreased by at least 66%. This is consistent with findings reported by a previous study that after less than 15 min of pre-oxidation with 2 mg/L O_3_, the inactivation efficiency for viable *Synedra* sp. cells reached 83% [47]. It can be concluded that low-dose O_3_ pre-treatment is able to effectively inactivate high-density *Synedra* sp.

Combining the results from this and the previous section, it is evident that for both *M. aeruginosa* and *Synedra* sp., the inactivation rate was highest within the first 10 min of O_3_ application for both the 0.5 mg/L and 1.5 mg/L doses. The photosynthetic inactivation effect tended to diminish with further increase in reaction time. This phenomenon is closely related to the inherent instability and rapid decomposition of O_3_ in aqueous solutions. O_3_ is known as a highly potent oxidant but also degrades very rapidly. A previous study reported that initial O_3_ concentrations of 1.073 mg/L and 1.426 mg/L decomposed completely after approximately 100 s and 300 s of reaction, respectively [48].

### 3.3. Enhanced Integrated Process: Micro-Acidified Low-Dose KMnO_4_-PAC-Conventional Treatment

Previous research has established that conventional water treatment processes alone offer limited control over the DBPs-FP arising from the typical algal species found in WTPs of Chongqing. Building upon the inactivation results for KMnO_4_ and O_3_ pre-oxidation detailed in the preceding sections, this section proposes and evaluates two enhanced integrated control strategies for more precise control of DBPs-FP from *M. aeruginosa* and *Synedra* sp.: (1) Micro-acidified low-dose KMnO_4_–PAC–conventional treatment, and (2) Low-dose O_3_ pre-oxidation–conventional treatment–O_3_–BAC. This study referenced the maximum allowable limits for DBPs stipulated in the Chinese Standards for Drinking Water Quality (GB 5749-2022) [49] and the World Health Organization (WHO) Guidelines for Drinking-water Quality (Fourth Edition) [50]. The detected types of DBPs and their corresponding regulatory limits are summarized in Table 1.

**Figure 7:**
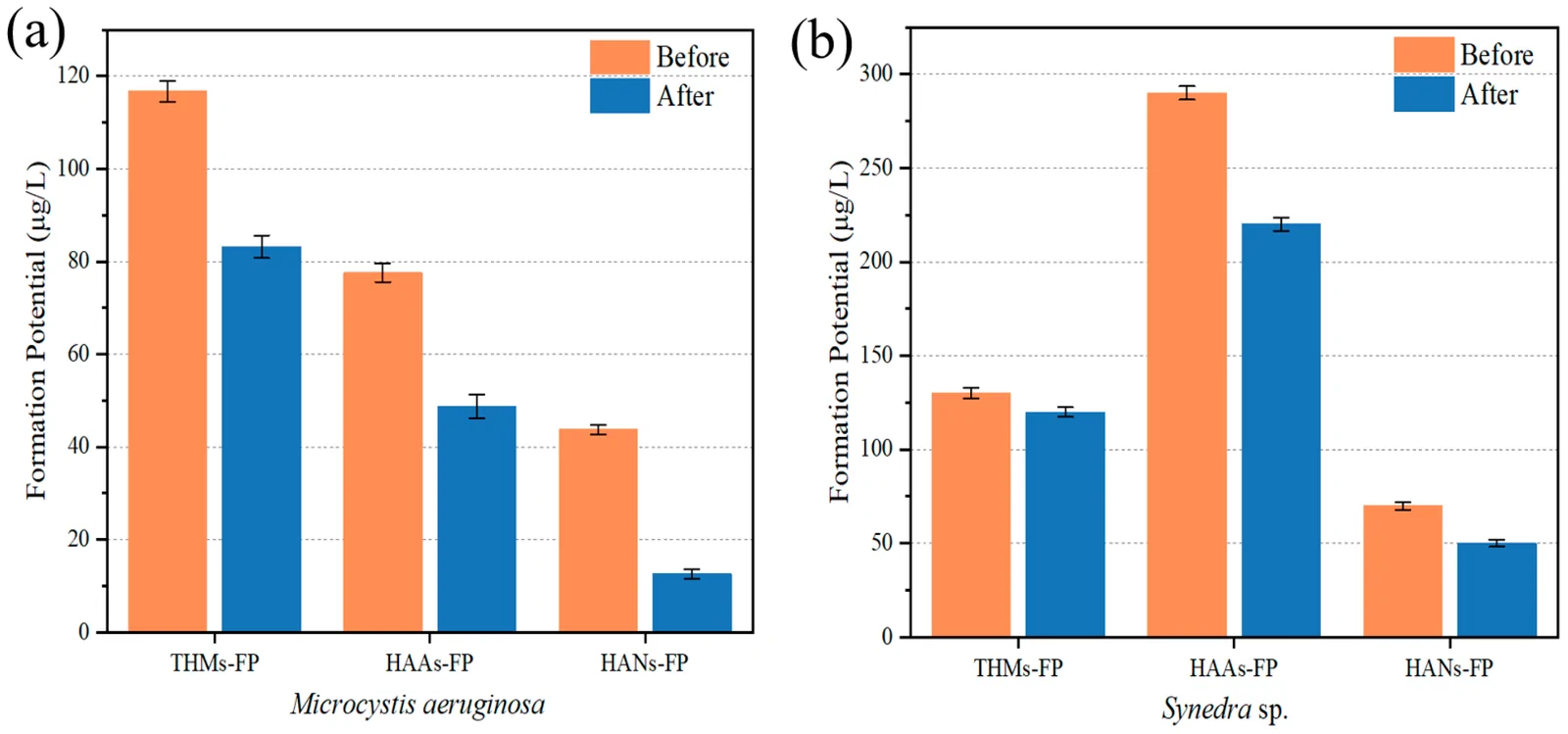
Control of DBPs-FP from (**a**) *M. aeruginosa* and (**b**) *Synedra* sp. by conventional water treatment process.

Conventional treatment alone showed a baseline level of DBPs-FP control from both algal species (Figure 7). For *M. aeruginosa*, the removal rates were 28.8% for THMs-FP, 37.2% for HAAs-FP, and 71.2% for HANs-FP. The corresponding post-treatment concentrations were 83.2 μg/L (THMs-FP), 48.7 μg/L (HAAs-FP), and 12.6 μg/L (HANs-FP). For *Synedra* sp., the removal rates were lower: 15.0% (THMs-FP), 25.0% (HAAs-FP), and 18.4% (HANs-FP), yielding concentrations of 112.0 μg/L, 217.3 μg/L, and 49.4 μg/L, respectively. Critically, for both algal species after conventional treatment, the THMs-FP concentrations exceeded the standard limit (60 μg/L), and the HAAs-FP values were close to or exceeded their limit (50 μg/L). These findings clearly demonstrate the inadequacy of conventional processes alone in controlling DBPs-FP to safe levels for this high-algae-containing water, necessitating the implementation of enhanced or emergency treatment strategies.

The PAC adsorption time (0.5 h, 1 h and 2.5 h) and corresponding dosage levels (Low, Medium and High) were established based on considerations of the hydraulic transport time from intake points at typical Chongqing WTPs and the requirements of GB50013-2018 “Outdoor Water Supply Design Standard” [51]. The detailed combination of adsorption time and dosages was set as shown in Table 2 below.

#### 3.3.1. Control of *M. aeruginosa*-Derived DBPs

**Figure 8:**
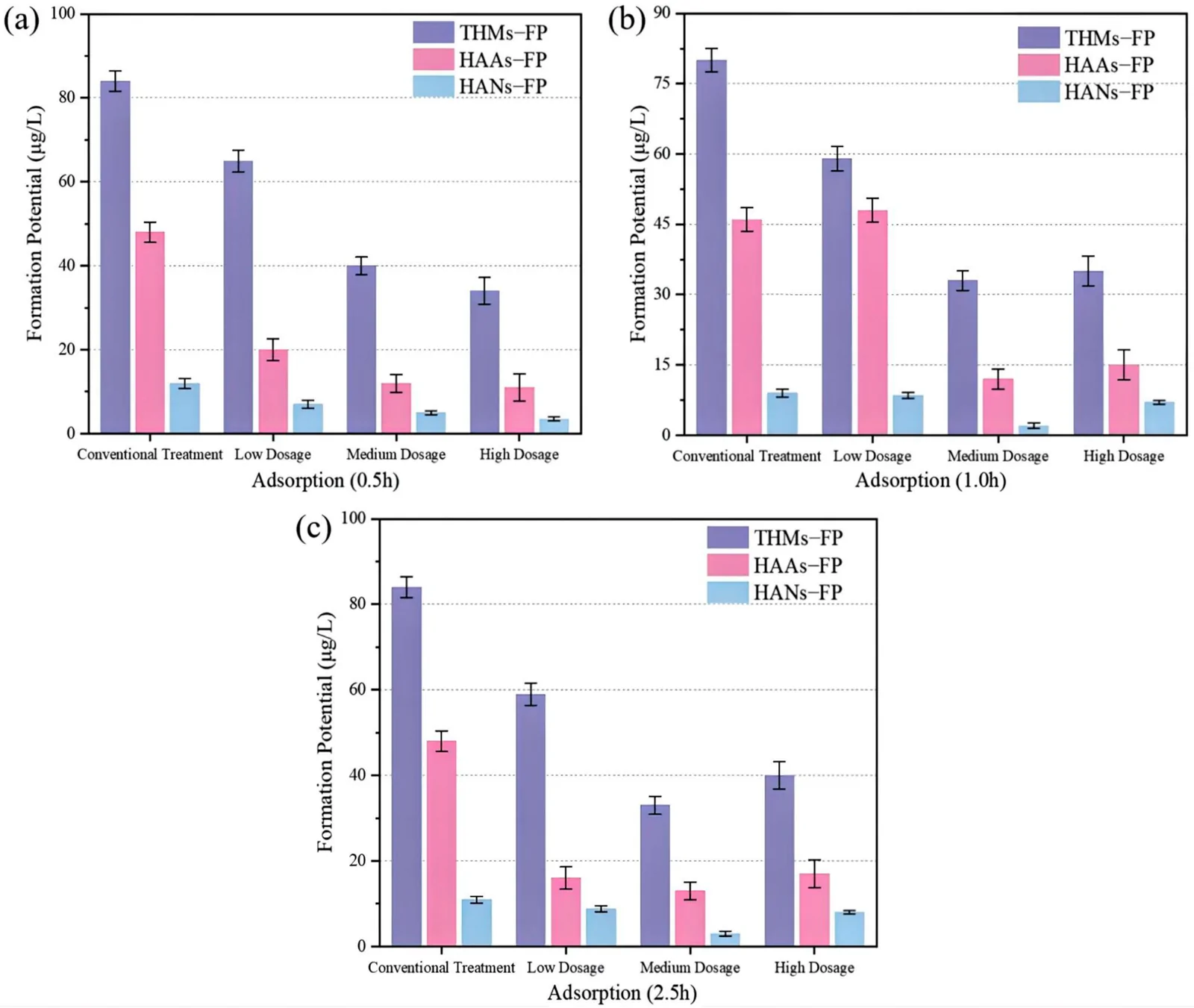
Control efficacy of slightly acidified and low-dose KMnO_4_–PAC–conventional water treatment processes on DBPs-FP from *M. aeruginosa* under varying PAC adsorption time (**a**) 0.5 h; (**b**) 1.0 h; (**c**) 2.5 h.

The control effects on the three target DBP classes under the integrated micro-acidified KMnO_4_–PAC–conventional process are shown in Figure 8. The results indicate that under all tested PAC adsorption time/dosage conditions, the removal rates for THMs-FP, HAAs-FP, and HANs-FP were significantly higher than those achieved by conventional treatment alone, with removal rates enhanced by 58–76%. The optimal performance was observed with a PAC dosage of 60 mg/L and an adsorption time of 0.5 h, which achieved the lowest DBPs-FP levels: THMs-FP at 34.3 μg/L, HAAs-FP at 11.9 μg/L, and HANs-FP at 3.9 μg/L.

In summary, testing across different PAC adsorption times and dosages confirmed that the emergency integrated process involving micro-acidified low-dose KMnO_4_ followed by PAC adsorption and conventional treatment significantly enhanced the overall control of *M. aeruginosa*-derived DBPs-FP, improving removal rate by 58–76% compared to conventional treatment alone. Even when considering the potential background level of DBPs-FP in the final effluent of Chongqing WTPs, the concentrations of THMs-FP, HAAs-FP, and HANs-FP detected after this emergency process under all PAC conditions at their respective optimal dosages remained entirely below the maximum allowable limits, which are set by the Chinese drinking water standards. It should be noted that the condition of 60 mg/L PAC for 0.5 h yielded the highest removal efficiency under ideal laboratory conditions. However, considering practical constraints such as cost, sludge handling, and the existing hydraulic residence time of Chongqing WTPs (typically >2.5 h from intake to plant), the condition of 20 mg/L for >2.5 h is recommended as the technically and economically optimal operational parameter for full-scale implementation. Therefore, the determined optimal operational parameters for this emergency process targeting *M. aeruginosa* control are: KMnO_4_ pre-oxidation dose of 1.5 mg/L at pH = 5.0; PAC adsorption time > 2.5 h and dosage of 20 mg/L; and Fe^3+^ coagulant dosage of 10 mg/L within the conventional treatment stage.

#### 3.3.2. Control of *Synedra* sp.-Derived DBPs

**Figure 9:**
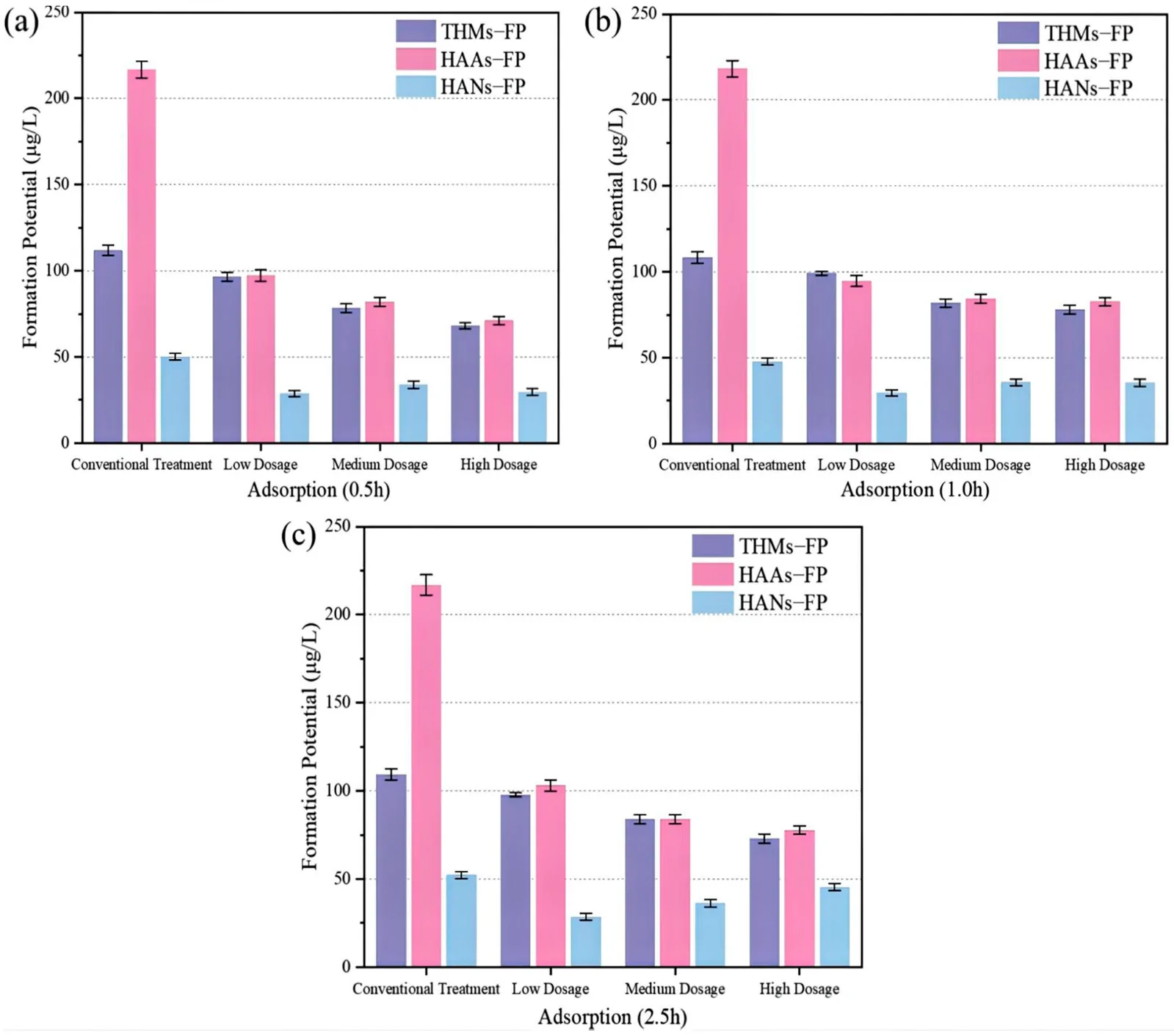
Control efficacy of slightly acidified and low-dose KMnO_4_–PAC–conventional water treatment process on DBPs-FP from *Synedra* sp. under varying PAC adsorption time (**a**) 0.5 h; (**b**) 1.0 h; (**c**) 2.5 h.

The same integrated processes (micro-acidified low-dose KMnO_4_–PAC– conventional treatment) were applied to control *Synedra* sp.-derived DBPs-FP. The experimental results, as depicted in Figure 9, reveal that the control efficiency of *Synedra* sp. DBPs by integrated processes was relatively limited. The highest PAC dosages provided the best control for THMs-FP and HAAs-FP, yielding concentrations of 67.8 μg/L and 71.1 μg/L (0.5 h), 77.4 μg/L and 82.9 μg/L (1 h), and 72.7 μg/L and 77.8 μg/L (2.5 h), respectively. However, even under these optimal dosage conditions, the levels of all three DBP classes still exceeded the stipulated regulatory limits. Based on the well-known molecular-weight adsorption preference of PAC and the documented EOM characteristics of diatoms, it is reasonable to infer that the limited control of *Synedra* sp.-derived DBPs is attributable to their predominantly low molecular weight compounds [30]. The extracellular organic matter (EOM) released by *Synedra* sp. during its logarithmic growth phase is predominantly composed of small molecular weight compounds (1–100 kDa). However, PAC exhibits relatively lower adsorption efficiency for organic substances with molecular weights below approximately 500 Da, resulting in poor removal of the specific diatom-derived precursor. This species-dependent effect extends Xie et al.’s findings that KMnO_4_ preserves cell integrity but cannot compensate for the inefficiency of PAC on diatom-derived small-molecular-weight precursors [52].

### 3.4. Emergency Process: Low-Dose O_3_ Pre-Oxidation–Conventional Treatment–O_3_–BAC

This section systematically evaluated the control efficiency of an integrated emergency process, i.e., Low-dose O_3_ Pre-oxidation (1.5 mg/L, 15 min)–conventional Treatment–O_3_–BAC (Post O_3_: 2.0 mg/L, 5 min; BAC column contact time: 15 min), targeting the DBPs-FP from both *M. aeruginosa* and *Synedra* sp. The results demonstrated a remarkable algal species-dependent effectiveness of this integrated treatment process.

#### 3.4.1. Control of *M. aeruginosa*-Derived DBPs

**Figure 10:**
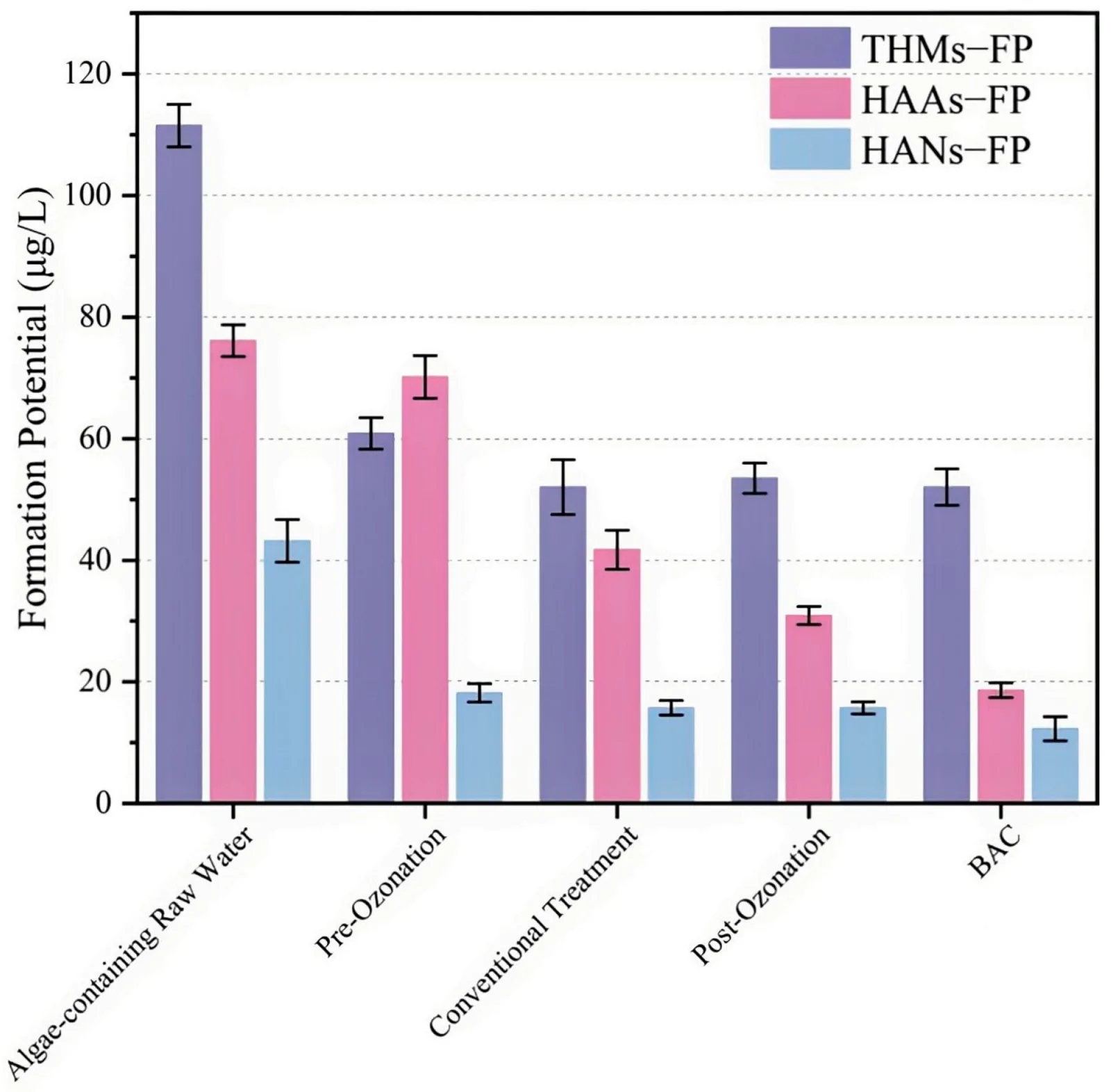
Control performance of low-dosage O_3_ pre-oxidation–conventional water treatment–O_3_–BAC processes on DBPs-FP from *M. aeruginosa*.

The performance of this integrated process in controlling *M. aeruginosa*-derived DBPs-FP is presented in Figure 10. The process provided a moderate degree of control over all DBP classes. The final effluent DBPs-FP concentrations were reduced to minimums of 52.4 μg/L for THMs-FP, 19.2 μg/L for HAAs-FP, and 13.0 μg/L for HANs-FP, with each overall removal rate across the entire process exceeding 50%. When considering the potential background DBPs-FP in a full-scale plant effluent, the THMs-FP concentration level would be controlled near the regulatory limit, while the HAAs-FP and HANs-FP concentrations would fall below their respective limits. According to the relative contribution of each individual unit process within the integrated scheme to *M. aeruginosa* DBPs-FP control, it reveals that O_3_ pre-oxidation accounted for 86% of the total THMs-FP removal, corresponding to a concentration reduction of 45.8%. In addition, the conventional treatment process made the largest contribution to HAAs-FP control (50% contribution, 37.5% concentration reduction), and O_3_ pre-oxidation was also the most effective unit for HANs-FP control (82% contribution, 57.6% concentration reduction). The standalone post-O_3_ process showed negligible effect on THMs-FP and HANs-FP but contributed to some removal of HAAs-FP. The incorporation of the BAC stage following post-O_3_ resulted in significant additional control for all three DBP classes. This can be explained by the mechanism of the O_3_–BAC process, in which ozonation could break down large and refractory organic molecules into smaller and more biodegradable intermediate compounds [53]. This oxidative transformation greatly improves the biodegradability of the organic matter, making it more amenable to subsequent removal via adsorption and biodegradation on the biologically active carbon filter [54]. The research made by Xu et al. also supports the conclusion that a pre-O_3_–conventional treatment–O_3_–BAC process achieved approximately 70% THMs-FP removal and 50% HAAs-FP removal from Huangpu River source water in China [31].

In conclusion, the low-dose O_3_ pre-oxidation–conventional treatment–O_3_-BAC processes offer a moderate level of controlling *M. aeruginosa*-derived DBPs-FP. However, the concentration of THMs-FP in the final effluent remains close to the standard limit, presenting a potential risk of non-compliance and indicating that it may not reliably achieve safe drinking water levels for this parameter when treating *M. aeruginosa*-laden water. Therefore, this specific integrated process is not recommended for emergency control of typical *M. aeruginosa*-derived DBPs in water treatment plants.

#### 3.4.2. Control of *Synedra* sp.-Derived DBPs

**Figure 11:**
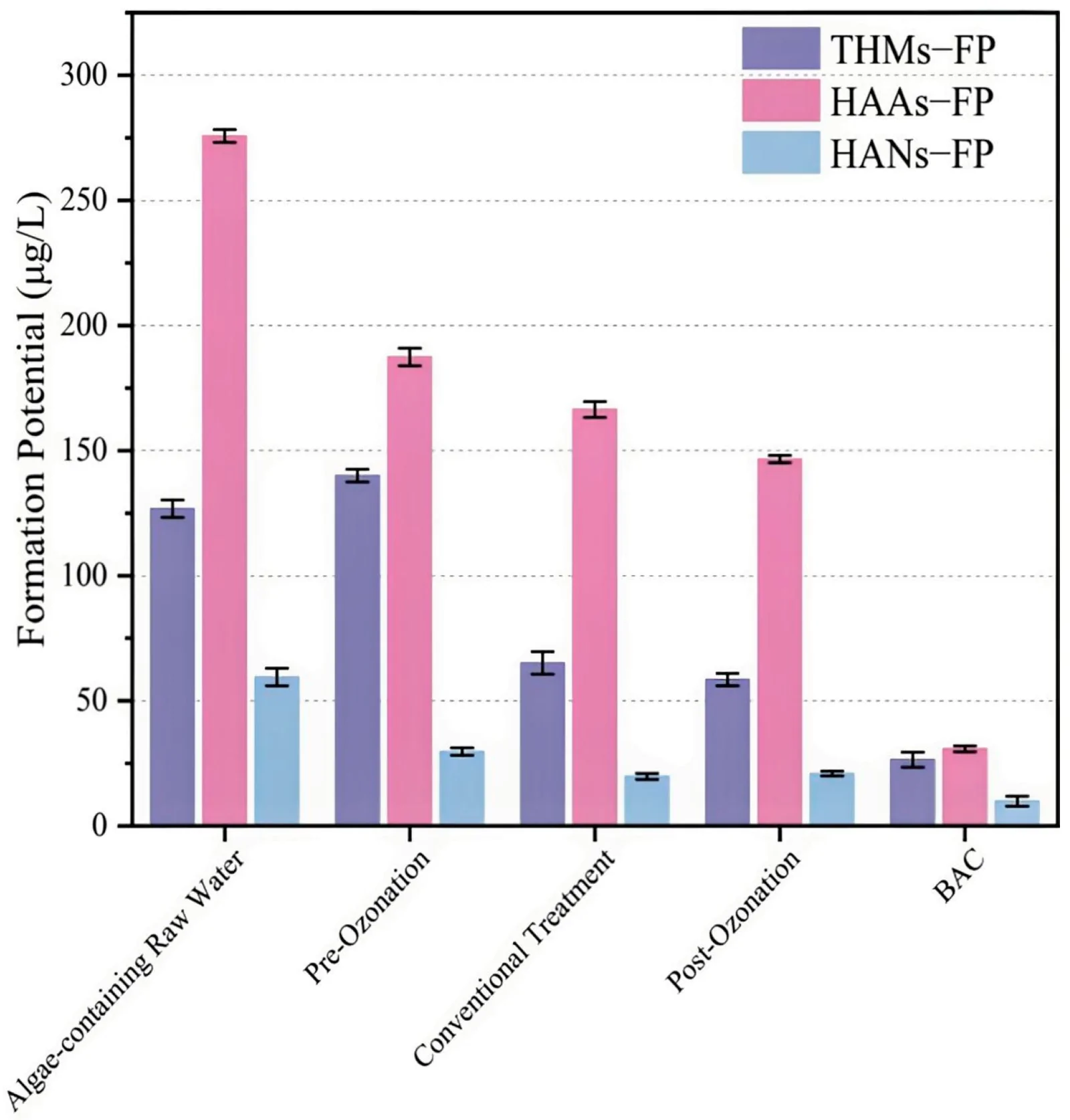
Control performance of low-dose O_3_ pre-oxidation–Conventional water treatment–O_3_–BAC processes on DBPs-FP from *Synedra* sp.

In contrast, the same integrated process proved highly effective for controlling *Synedra* sp.-derived DBPs-FP, as illustrated in Figure 11. The process achieved remarkable removal, with final effluent concentrations reduced to minimums of 27.6 μg/L for THMs-FP, 31.4 μg/L for HAAs-FP, and 10.5 μg/L for HANs-FP. The overall removal rates for all three DBP classes were exceptionally high, reaching around 80%. Even accounting for background DBPs-FP in the plant effluent, the generated levels of THMs-FP, HAAs-FP, and HANs-FP would all remain comfortably below their respective regulatory limits.

The main reason for this efficient control lies in the synergistic interplay between the unit processes. The conventional treatment stage played a dominant role in controlling THMs-FP (74% contribution), effectively compensating for a short-term increase in THMs-FP observed after the O_3_ pre-oxidation step alone (which showed a negative contribution of 13%). This concentration increase could be due to O_3_ pre-oxidation disrupting algal cells and releasing intracellular organic matter (IOM), which may contain THM precursors [55]. The synergistic mechanism of the O_3_–BAC process can be further elucidated by comparison with established studies. Chen et al. demonstrated that the optimal process combination for controlling DBPs and their precursors is pre-oxidation (with either ozone or potassium permanganate) coupled with conventional treatment and O_3_–BAC [56]. Recent studies on O_3_–BAC technology confirm that this process effectively removes algae, organic matter, and DBPs precursors [57,58]. Importantly, ozonation alone does not mineralize organic matter but rather breaks down large molecules into smaller, more biodegradable compounds—a transformation that can actually increase the formation potential of some nitrogenous DBPs [59].

However, the subsequent BAC filtration removes these ozonation-transformed, readily biodegradable substances, achieving a synergistic reduction in overall DBPs-FP. This synergy is particularly effective for *Synedra* sp. because its EOM, being predominantly small-molecular-weight, is more readily biodegraded after ozonation. In contrast, the high-molecular-weight polysaccharide sheath of *M. aeruginosa* is less susceptible to both ozonation and subsequent biodegradation, explaining the poor performance of the same O_3_–BAC train for this species. Plummer et al. similarly observed that O_3_ pre-oxidation could cause the release of EOM from cyanobacterial cells, increasing the concentration of THM precursors [60,61]. The O_3_ pre-oxidation and subsequent BAC process acted synergistically to degrade the small molecular weight EOM (1–100 kDa) characteristic of *Synedra* sp., jointly controlling HAAs-FP (36% and 48% contribution) and HANs-FP (59% and 21% contribution). The BAC filter, with a contact time of 15 min, efficiently adsorbed the ozonation-transformed, more readily biodegradable organic substances. Therefore, for the control of *Synedra* sp.-derived DBPs, the adoption of this integrated O_3_-based emergency treatment process is strongly recommended. The optimal technical parameters derived from this study for controlling *Synedra* sp. DBPs-FP are summarized in Table 3 below.

### 3.5. Limitations

While this study delivers practical insights for algal bloom and associated DBPs-FP control, a few methodological boundaries should be noted. First, we used single-species laboratory cultures in a synthetic matrix. This was a deliberate choice to isolate algal precursor effects from confounding natural organic matter and microbes, a standard practice in mechanistic DBP-FP studies. Nevertheless, natural blooms are multi-species and contain background constituents that may influence oxidant demand; field validation with actual source waters is thus encouraged.

In addition, we did not directly characterize algal organic matter (e.g., DOC, UV_254_, molecular weight fractions). Our mechanistic explanations are therefore indirect, derived from DBP-FP removal contribution patterns across treatment units and supported by established literature. This approach is fully adequate for engineering optimization, which was our primary goal, but direct AOM analysis would be valuable to confirm the proposed transformation pathways in future studies. Moreover, the PHYTO-PAM parameters measure photosynthetic activity, not cell lysis or membrane integrity. We acknowledge that oxidation-induced lysis may release additional precursors, but our data do not distinguish this from reversible inhibition. Complementary assays (e.g., viability staining, flow cytometry) would help resolve this, and we recommend them for future study. In addition, our DBPs-FP values represent worst-case potential formation under exaggerated chlorination, not actual finished-water concentrations. Comparing them with regulatory limits is intentionally conservative and provides a robust safety margin for process design. The actual plant effluents would be expected to show lower levels.

Although these limitations qualify the scope of our conclusions, they do not reduce the main contributions of this study, including species-specific performance evaluation of integrated treatment trains, identification of differential responses between *M. aeruginosa* and *Synedra* sp., and directly applicable recommendations for Chongqing WTPs. We hope this work stimulates field-based and characterization-focused subsequent studies to refine the theoretical understanding and operational guidelines.

## 4. Conclusions

This study focused on the control of typical dominant bloom-forming algal species in water treatment plants of Chongqing City through pre-oxidation. The photosynthetic inactivation of *M. aeruginosa* and *Synedra* sp. and the control of their associated DBPs-FP using pre-oxidation with potassium permanganate (KMnO_4_) and ozone (O_3_) were investigated. Based on the distinct inactivation profiles of the different algal species, targeted integrated water treatment processes were proposed for the effective control of DBPs-FP concentration for each dominant species, accompanied by the determination of relevant optimal operational parameters.

For the control of *M. aeruginosa*, pre-oxidation with a micro-acidified (pH = 5.0) low dose of KMnO_4_ (1.5 mg/L) for 30 min significantly inactivated algal cells and disrupted their photosynthetic activity. For comprehensive control of *M. aeruginosa*-derived DBPs, the optimal strategy identified was the integrated sequence: Micro-acidified KMnO_4_ (1.5 mg/L)–PAC (with dosages of 20–60 mg/L, particularly effective for adsorption time > 0.5 h)–conventional processes. This scheme proved highly effective in reducing DBPs-FP, with overall removal rates of 58–76% for THMs-FP, >75% for HAAs-FP, and >70% for HANs-FP compared to conventional treatment alone (see Figure 8 and Section 3.3.1). Under the optimal conditions (PAC 20 mg/L, adsorption time ≥ 2.5 h), the final effluent concentrations of THMs-FP, HAAs-FP and HANs-FP were reduced to below the Chinese regulatory limits.

For the control of *Synedra* sp., Low-dose O_3_ pre-oxidation (1.5 mg/L, 15 min) efficiently inactivated *Synedra* sp. cells. For controlling DBPs-FP originating from *Synedra* sp., the implementation of the following integrated process was recommended: O_3_ pre-oxidation (1.5 mg/L)–conventional treatment–O_3_-BAC advanced treatment (incorporating Post-O_3_ at 2.0 mg/L for 5 min and a BAC column contact time of 15 min). This process configuration ensures simultaneous and effective control of THMs-FP, HAAs-FP, and HANs-FP, with overall removal rates reaching approximately 80% for all three DBP classes (see Figure 11 and Section 3.4.2). The final effluent concentrations were reduced to as low as 27.6 μg/L for THMs-FP, 31.4 μg/L for HAAs-FP, and 10.5 μg/L for HANs-FP, all comfortably below the respective guideline limits.

This study provides valuable insights and practical engineering references for the improvement and optimization of conventional water treatment plant processes, specifically targeting algal photosynthetic inactivation and the associated challenge of controlling algae-derived DBPs. The findings highlight the remarkable efficiency of the O_3_–BAC process for controlling algae-derived DBPs, suggesting that large water treatment plants in regions prone to relevant algal blooms should prioritize the incorporation of this technology to enhance the synergistic removal of multiple DBP classes. It is important to note that the current experiments were conducted using single-species algal cultures. Future research should aim to more accurately simulate the complex water quality conditions encountered during actual algal blooms, which often involve mixtures of multiple algal species, to further refine and optimize emergency control strategies for diverse algal bloom scenarios.

## Figures and Tables

**Table 1:** Types of DBPs Detected and Allowable Limits.

DBPs Type	GB 5749-2022 Limit (μg/L)	WHO Guideline Limit (μg/L)
Trihalomethanes (THMs)	60	-
Haloacetic Acids (HAAs)	50	-
Haloacetonitriles (HANs)	-	20

**Table 2:** Combination of PAC adsorption time and dosage.

Adsorption Time (h)	Low Dosage (mg/L)	Medium Dosage (mg/L)	High Dosage (mg/L)
0.5	30	40	60
1	15	20	30
2.5	15	20	30

**Table 3:** Optimal technical parameters of low-dose O_3_ pre-oxidation–conventional water treatment–O_3_–BAC processes for controlling DBPs-FP from *Synedra* sp.

Process Stage	Specific Parameters
O_3_ Pre-oxidation	Dose: 1.5 mg/L; Contact Time: ≥15 min
Conventional Treatment	Coagulant: 10 mg/L (as Fe^3+^)
Post-O_3_ Treatment	Dose: 2.0 mg/L; Contact Time: 5 min
BAC	Contact Time: 15 min

## Data Availability

The original contributions presented in this study are included in the article. Further inquiries can be directed to the corresponding author.

## Supplementary Materials

The following supporting information can be downloaded at: https://www.mdpi.com/article/10.3390/toxics14090826/s1, Text S1: The detailed procedures for determining targeted DBPs-FP; Text S2: The determination and computation of typical photosynthetic activity parameters; Table S1: BG11 medium formulae; Table S2: AGP medium formulae; Figure S1: The representative GC-ECD chromatograms of THMs; Figure S2: The representative GC-ECD chromatograms of HAAs; Figure S3: The representative GC-ECD chromatograms of HANs; Figure S4: Calibration curve of THMs; Figure S5: Calibration curve of HAAs; Figure S6: Calibration curve of HANs.
