# Supplementary material for: Control of Algae-Induced Disinfection By-Products Formation by Pre-Oxidation for Typical Algal Species in Drinking Water Treatment Plants of Chongqing, China
Source: Toxics. 2026 Sep 17;14(9):826. doi: 10.3390/toxics14090826 (PMC13611760; doi:10.3390/toxics14090826)
Supplement: Supplementary file 1 [file toxics-14-00826-s001.zip › toxics-4487045-supplementary.pdf]

Supplementary materials

Control of Algae-Induced Disinfection By-Products Formation by Pre-oxidation for Typical Algal Species in Drinking Water Treatment Plants of Chongqing, China

Xiangyu Chen[1]*, Shuhan Xia[1], Hao Zhong[1], Dan Gong[2]，Haibing Cong[1]

1. *College of Environmental Science and Engineering, Yangzhou University, Yangzhou 225127, Jiangsu, China*
2. *Key Laboratory of the Three Gorges Reservoir Regions Eco-Environment, Ministry of Education, Chongqing University, Chongqing 400045, China*

*Corresponding author, e-mail: (xiangyuchen@yzu.edu.cn)

Text S1. The detailed procedures for determining targeted DBPs-FP

For the determination of disinfection by-product formation potential (DBPs-FP) using gas chromatography with electron capture detection (GC-ECD), the extraction and derivatization procedures followed USEPA methods 551.1 for trihalomethanes (THMs) and haloacetonitriles (HANs) and USEPA method 552.3 for haloacetic acids (HAAs). Specifically, for THMs and HANs, a 40 mL sample was extracted with methyl tert-butyl ether (MTBE) containing 1,2-dibromopropane as an internal standard after salting out with anhydrous sodium sulfate, and the organic phase was dehydrated and directly injected into the chromatograph. For HAAs, a 30 mL sample was acidified to pH <0.5, extracted with MTBE, and then derivatized with acidic methanol (10% sulfuric acid in methanol) at 50 °C for 2 h; after cooling and washing with saturated sodium bicarbonate, the organic layer was injected.

The chromatographic separation was performed on a Shimadzu GC-2010plus instrument equipped with an ECD and a DB-5 capillary column (30 m×0.25 mm i.d.×0.25 μm film thickness), using nitrogen as both carrier gas at 1.0 mL/min and make-up gas at 30 mL/min. The injector was operated in splitless mode at 200 °C with an injection volume of 1 μL, and the oven temperature was programmed from 40 °C (held for 2 min) to 100 °C at 10 °C/min, then to 240 °C at 20 °C/min (held for 1 min), while the detector temperature was maintained at 280 °C. Calibration was performed using external standards with internal standard correction, employing standard solutions of four THMs, four HAAs, and two HANs purchased from Aladdin, with calibration ranges of 0.5–200 μg/L for THMs, 1.0–200 μg/L for HAAs, and 0.5–100

μg/L for HANs, all yielding correlation coefficients (R^2) greater than 0.995. The method detection limits were 0.3, 0.5, and 0.2 μg/L for THMs, HAAs, and HANs, respectively, and the corresponding limits of quantification were 1.0, 2.0, and 1.0 μg/L.

Text S2. The determination and computation of typical photosynthetic activity parameters

1. A series of representative photosynthetic parameters, consisting of Y (dimensionless), α (μmol electrons $m^{-2}s^{-1}$/μmol photons $m^{-2}s^{-1}$) and rETRmax (μmol $m^{-2}s^{-1}$), along with the concentration of chlorophyll-a (μg L^{-1}) were determined by a Phytoplankton Analyzer (PHYTO-PAM, Walz, Germany). A 2 mL sample was taken with a pipette and put into the measuring cup. After 15 min dark adaptation, these typical parameters were automatically derived in real-time by the built-in software of the PHYTO-PAM during each measurement session.

The effective quantum yield of PS II (Y) was calculated from the measured fluorescence value using the following formula: $Y = (F_{m'} - F_t) / F_{m'}$, where $F_{m'}$ is the maximum fluorescence yield under light-adapted conditions and F_t is the steady-state fluorescence signal. The initial slope (α) and maximum relative electron transport rate (rETRmax) were derived from fitting the rapid light curve (RLC) using the formula: $rETR = rETR_{max} \times (1 - e^{-\alpha} \times PAR/rETR_{max})\ e^{-\beta} \times PAR/rETR_{max}$. Where α is the initial slope of the RLC curve, which reflects the light use efficiency, and β is the photoinhibition parameter. $rETR_{max}$ represents the maximum potential relative electron

transfer rate without photoinhibition, and rETR represents the relative electron transfer rate [1]. The photosynthetically active radiation (PAR) of actinic light was increased by 100 μmol/(s·m^2) every 10 s from 1 μmol/(s·m^2) up to 1000 μmol/(s·m^2) to obtain the rapid light curve (RLC) by fitting the formula.

2. Chlorophyll-a concentration was directly determined by the PHYTO-PAM analyzer through in vivo fluorescence measurements. The method is based on the measurement of chlorophyll-a fluorescence emitted from living algal cells under controlled light conditions, without destroying the cells or extracting pigments . The underlying principle is that the minimum fluorescence yield (F_0) in dark-adapted samples is proportional to the chlorophyll-a concentration in the sample. A linear relationship exists between F_0 and Chlorophyll-a concentration over a wide range (approximately 0–500 μg/L), which is the basis for quantification.

For the measurement procedure, a 2 mL aliquot of well-mixed algal suspension was transferred into the PHYTO-PAM measuring cuvette. The sample was dark-adapted for 15 min at room temperature to ensure complete oxidation of all PSII reaction centers. The dark-adapted sample was then illuminated with weak measuring light (wavelengths: 470, 520, 645, and 665 nm), and the emitted fluorescence was detected by the photodiode of instrument. Four excitation wavelengths were used to differentiate phytoplankton groups (cyanobacteria, green algae, diatoms and dinoflagellates) based on their pigment-specific absorbance spectra. The minimal fluorescence (F_0) was recorded for each measuring channel, and the PhytoWin software automatically computed the chlorophyll-a concentration using the following

relationship: $\text{Chl a} = F_0 / (k \times \text{spectrum})$

Where Chl a is chlorophyll-a concentration (μg/L), F_0 is minimum fluorescence yield in the dark-adapted state, k is instrument-specific calibration factor (determined by reference spectra), and spectrum is reference spectrum of the target algal group.

Table S1 BG11 medium formulae

#	Component	Dosage	Stock solution concentration
1	$NaNO_3$	10 mL/L	5 g/100mL dH_2O
2	K_2HPO_4	10 mL/L	1.5 g/100mL dH_2O
3	$MgSO_4·7H_2O$	10 mL/L	1 g/100mL dH_2O
4	$CaCl_2·2H_2O$	10 mL/L	0.7 g/100mL dH_2O
5	Citric acid	10 mL/L	0.5 g/100mL dH_2O
6	Ferric ammonium citrate	10 mL/L	1 g/100mL dH_2O
7	Na_2EDTA	10 mL/L	0.5 g/100mL dH_2O
8	Na_2CO_3	10 mL/L	1.0 g/500mL dH_2O
9	A5 (Trace mental solution)	1 mL/L	
	H_3BO_3		2.86 g/L dH_2O
	$MnCl_2·4H_2O$		1.86 g/L dH_2O
	$ZnSO_4·7H_2O$		0.22 g/L dH_2O
	$Na_2MoO_4·2H_2O$		0.39 g/L dH_2O
	$CuSO_4·5H_2O$		0.08 g/L dH_2O
	$Co(NO_3)_2·6H_2O$		0.05 g/L dH_2O

Table S2 AGP medium formulae

#	Component	Dosage	Stock solution concentration (mg/L)
1	$NaNO_3$	2 mL/L	25.5
2	K_2HPO_4	2 mL/L	1.04
3	$MgSO_4 \cdot 7H_2O$	2 mL/L	14.7
4	$CaCl_2 \cdot 2H_2O$	2 mL/L	4.41
5	$NaHCO_3$	2 mL/L	15
6	$MgCl_2$	2 mL/L	5.7
7	H_3BO_3	2 ml/L	186
	$MnCl_2$		264
	$ZnCl_2$		3.27
	$CoCl_2$		0.78
	$CuCl_2$		0.009
	$Na_2MoO_4 \cdot 2H_2O$		7.26
	$FeCl_3$		96
	$Na_2EDTA \cdot 2H_2O$		300

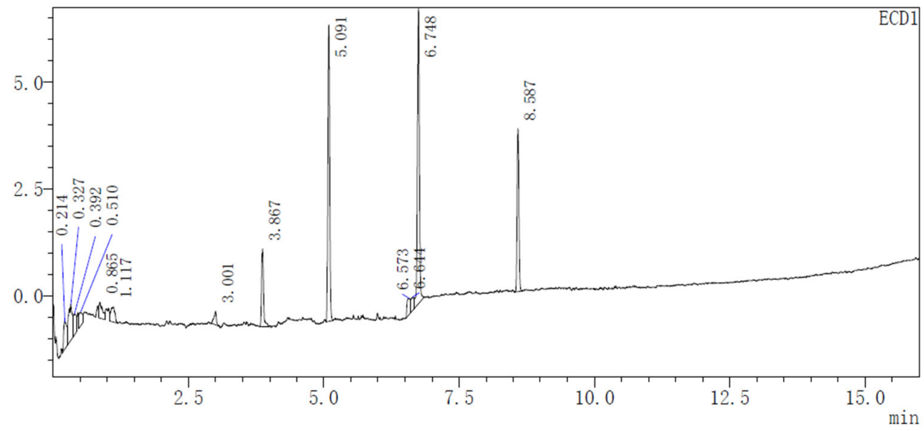

Figure. S1 The representative GC-ECD chromatograms of THMs

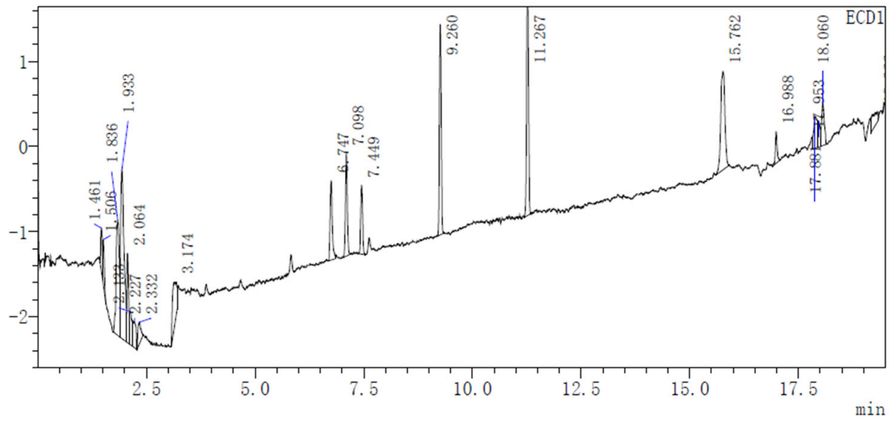

Figure. S2 The representative GC-ECD chromatograms of HAAs

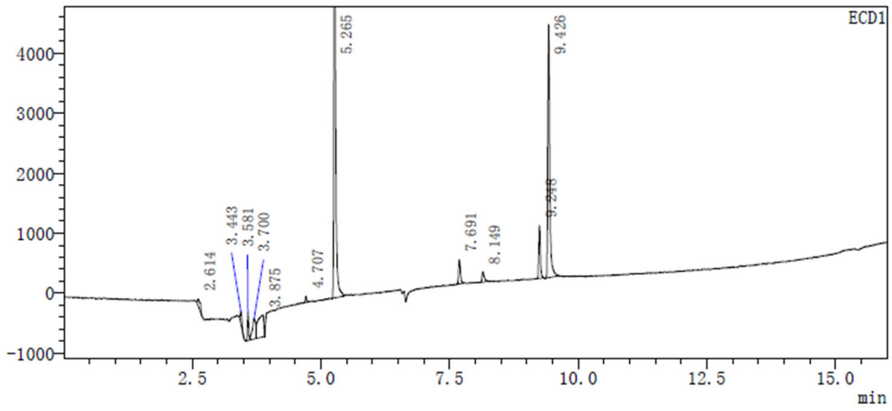

Figure. S3 The representative GC-ECD chromatograms of HANs

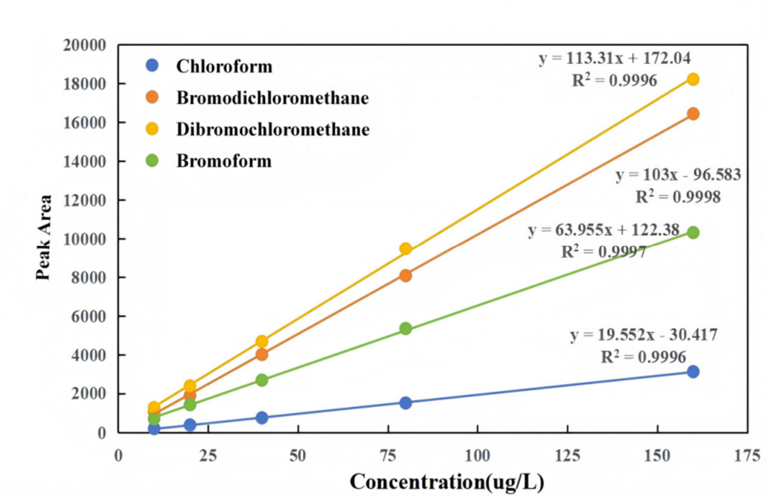

Figure. S4 Calibration curve of THMs

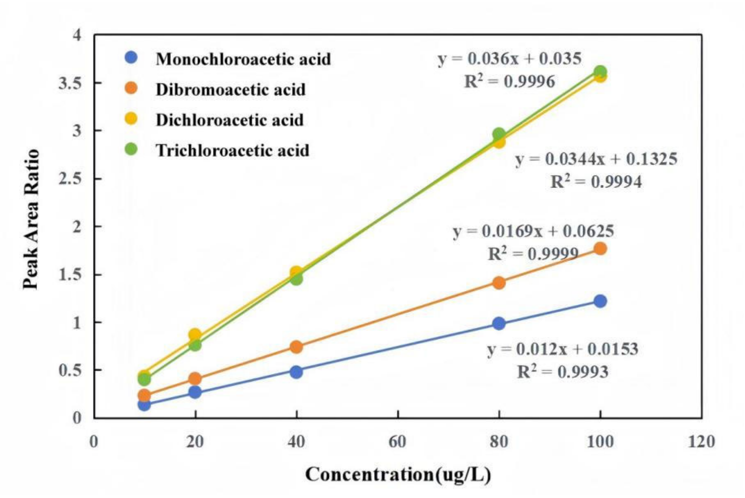

Figure. S5 Calibration curve of HAAs

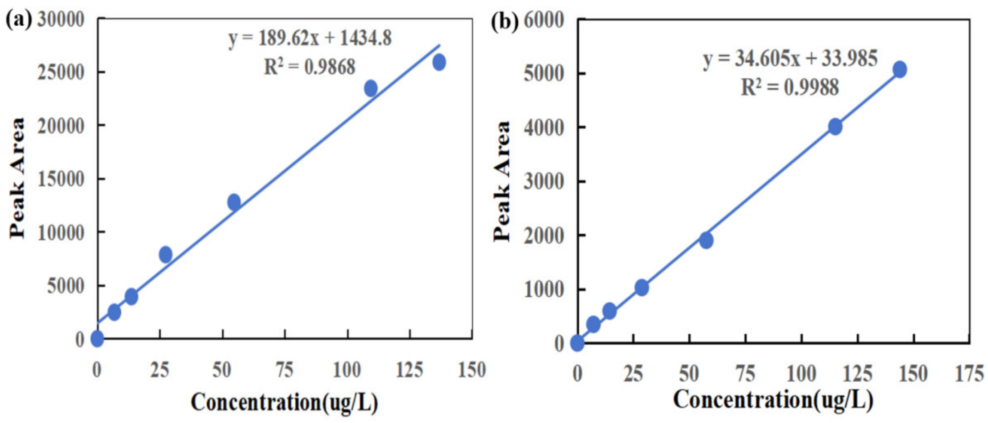

Figure. S6 Calibration curve of HANs (a) Dichloroacetonitrile (b) Trichloroacetonitrile

References

1. Ralph, P.J.; Gademann, R. Rapid light curves: A powerful tool to assess photosynthetic activity. *Aquat. Bot.* **2005**, *82*, 222-237.
